# Conserved mobile genetic contexts associated with *bla*_NDM-1_ and *bla*_NDM-5_in *Acinetobacter baumannii* isolated from two hospitals in Thailand

**DOI:** 10.7717/peerj.21848

**Published:** 2026-10-27

**Authors:** Thawatchai Kitti, Kritnarin Kaewphu, Akachai Khuekankaew, Chalermchai Boonlao, Supat Khongfak, Sutthirat Sitthisak

**Affiliations:** 1 Department of Oriental Medicine, Chiang Rai College, Chiangrai, Thailand; 2 Microbiology and Parasitology, Naresuan University, Phitsanulok, Thailand; 3 Department Clinical, Microbiology, Chiangrai Prachanukroh Hospital, Chiang Rai, Thailand; 4 Office of Disease Prevention and Control Region 3 Nakhon Sawan, Department of Disease Control, Ministry of Public Health, Nakhon Sawan, Thailand

**Keywords:** New Delhi metallo-β-lactamase, blaNDM-1, blaNDM-5, Acinetobacter baumannii, IS91

## Abstract

**Background:**

*Acinetobacter baumannii* has emerged as a major nosocomial pathogen due to its remarkable ability to develop resistance to multiple classes of antimicrobial agents. The emergence and dissemination of the *bla*_NDM_ gene among multidrug-resistant *A. baumannii* (MDRAB) are closely associated with mobile genetic elements (MGEs), which facilitate horizontal gene transfer and accelerate the spread of carbapenem resistance. This study investigated the distribution of *bla*_NDM_-positive *A. baumannii* isolates, characterized their antimicrobial resistance phenotypes and genetic determinants, and elucidated the genetic context surrounding *bla*_NDM_ alleles using whole-genome sequencing (WGS).

**Methods:**

A total of 286 clinical *A. baumannii* isolates collected from a tertiary hospital in Thailand were screened for metallo-*β*-lactamases encoding genes(*bla*_NDM_), OXA carbapenemase-encoding genes, IS*Aba1*, and IS*Aba125* using polymerase chain reaction (PCR). The *bla*_NDM_ variants were characterized by gene sequencing, and antibiotic susceptibility profiles were determined. Whole-genome sequencing (WGS) was performed on eight representative *bla*_NDM_-positive isolates, comprising four isolates from the present study and four isolates obtained from another hospital in Thailand.

**Results:**

Among the 286 *A. baumannii* isolates, 55 (19.23%) carried *bla*_NDM_. All *bla*_NDM_-positive *A. baumannii* isolates were identified as carbapenem-resistant *A. baumannii* (CRAB), and 90.91% were classified as MDRAB strains. The prevalence of *bla*_OXA-23_ and *bla*_OXA-58_ was 85.45% and 16.36%, respectively. The insertion sequences IS*Aba1* and IS*Aba125* were detected in 89.09% and 81.82% of the isolates, respectively. Sequence analysis revealed that 49 (89.09%) *bla*_NDM_-positive *A. baumannii* harbored *bla*_NDM-1_, whereas six (10.91%) showed sequence identity with *bla*_NDM-5_. WGS analysis of eight *bla*_NDM_-positive *A. baumannii* demonstrated variability in K-locus and OC-locus types. MLST analysis revealed that the four *bla*_NDM-1_-positive isolates belonged to three distinct sequence types (ST16, ST23, and ST213), while predominance of *bla*_NDM-5_-positive isolates were ST2. Comparative genomic analysis of the genetic environments surrounding the *bla*_NDM_ genes revealed highly conserved genetic organization, with strong synteny among isolates belonging to the same *bla*_NDM_ type.

**Conclusion:**

These findings demonstrate the emergence and persistence of multiple resistance determinants among *A. baumannii* isolates in Thailand, thereby underscoring the urgent need to strengthen antimicrobial stewardship program and infection prevention control strategies.

## Introduction

*Acinetobacter baumannii* has emerged as a significant clinical concern due to its remarkable capacity to develop resistance to multiple classes of antimicrobial agents and its frequent involvement in hospital-acquired outbreaks (Mukhopadhyay et al., 2024). The increasing prevalence of antibiotic-resistant *A. baumannii*, particularly multidrug-resistant (MDRAB) and extensively drug-resistant (XDRAB) phenotypes, has substantially limited therapeutic options and poses a major challenge to clinical management (Ibrahim et al., 2021). Data from the National Antimicrobial Resistance Surveillance Thailand (NARST) program consistently report a high prevalence of carbapenem-resistant *A. baumannii–calcoaceticus* complex isolates in intensive care units across tertiary-care hospitals nationwide. This data highlights the organism’s endemicity and considerable public health burden in Thailand.

Carbapenem-resistant *A. baumannii* (CRAB) has been designated as a critical priority pathogen by the World Health Organization, reflecting its substantial contribution to global morbidity and mortality (Thacharodi et al., 2024). The primary mechanism of carbapenem resistance in *A. baumannii* is the production of carbapenem-hydrolyzing enzymes, particularly Ambler class D *β*-lactamases (OXA-type carbapenemases), collectively known as carbapenem-hydrolyzing class D *β*-lactamases (CHDLs). The most prevalent CHDLs include the acquired OXA-23, OXA-24, and OXA-58-like enzymes, along with the intrinsic OXA-51-like family. Additionally, Ambler class B metallo-*β*-lactamases (MBLs), particularly New Delhi metallo-*β*-lactamase (NDM), have emerged as important contributors to carbapenem resistance (Castanheira, Mendes & Gales, 2023). Unlike OXA-type carbapenemases, *bla*_NDM_ genes are frequently associated with mobile genetic elements (MGEs), which facilitate horizontal gene transfer and promote the rapid dissemination of carbapenem resistance within healthcare settings (Wu et al., 2019). In Thailand, NDM-1-producing *A. baumannii* was first identified in clinical isolates collected during 2013–2014 and later reported in 2016 (Santimaleeworagun et al., 2016). Subsequently, NDM-5–producing *A. baumannii* has also been identified in both clinical specimens and hospital environments (Kitti et al., 2023). Insertion sequences play a pivotal role in the acquisition and dissemination of antimicrobial resistance determinants in *A. baumannii*. In particular, IS*Aba125* has been strongly implicated in mobilizing *bla*_NDM_ genes by providing a potent promoter that enhances transcriptional activity and therefore increases enzyme production (Kumari et al., 2025).

Although there have been increasing reports of *bla*_NDM_-harboring *A. baumannii* worldwide, longitudinal data describing its prevalence, genetic characteristics, and associated antimicrobial resistance determinants in Thailand remain limited. Furthermore, the genetic contexts surrounding *bla*_NDM_ alleles and their association with MGEs have not been comprehensively characterized. Therefore, this study aimed to investigate the temporal distribution of *bla*_NDM_-positive *A. baumannii* isolates collected from a tertiary hospital in Thailand between 2014 and 2024. Additionally, the study aimed to characterize their antimicrobial susceptibility profiles and resistance determinants and examine the genetic environments surrounding *bla*_NDM_ alleles using whole-genome sequencing (WGS). The association between *bla*_NDM_ genetic contexts and mobile genetic elements was also investigated to provide insight into the dissemination of carbapenem resistance in this clinical setting.

## Materials and Methods

### Bacterial isolation and species identification

A total of 286 non-duplicate clinical isolates of *Acinetobacter* spp. were collected in 2014, 2018, and 2024 from Chiangrai Prachanukroh Hospital, a 773-bed tertiary hospital that manages approximately 3,500 patient admission per day. All isolates were initially identified to the genus level using standard biochemical assays, including Gram staining, motility testing, catalase testing, oxidase testing, and Leeds Acinetobacter Agar (LAM) profiling. PCR amplification of the 16S rRNA and *rpoB* genes was performed, and species-level identification of *A. baumannii* was confirmed by detection of the intrinsic *bla*_OXA-51_ as previously described (Turton et al., 2006). The isolate was stored at −40 °C in Luria–Bertani (LB) broth (HiMedia, India) supplemented with 20% glycerol. The study protocol was approved by the Naresuan University Institutional Biosafety Committee (Approval No. NUIBC MI66-03-08) and the Naresuan University Institutional Human Ethics Review Board (Approval No. NUIBC MI67-01-01). The study was granted an exemption from human ethical review by the Naresuan University Institutional Review Board (NU-IRB) (Exemption Approval No. MHESI 0603.01.13(1)/NU-IRB 0789).

### Detection of antibiotic resistance genes and insertion sequences (IS*Aba1* and IS*Aba125*)

Polymerase chain reactions (PCR) assays were performed to detect metallo-*β*-lactamases encoding gene (*bla*_NDM_) and OXA type carbapenemases genes (*bla*_OXA-23_, *bla*_OXA-24_, *bla*_OXA-58_), following previously published protocol (Leungtongkam et al., 2018). Primer sequences are listed in Table S1. The presence of IS*Aba1* and IS*Aba125* gene were investigated in all *bla*_NDM_ positive strains using specific primers. PCR amplicons were separated by electrophoresis on 1% agarose gels and visualized under ultraviolet illumination.

### Sequencing of *bla*_NDM_ genes

The *bla*_NDM_ variants were determined by sequencing of PCR-amplified product obtained from all *bla*_NDM_ positive *A. baumannii* isolates. Amplification was performed using specific *bla*_NDM_ primer on crude bacterial cell lysates. The resulting PCR products were submitted for sequencing using a commercial amplicon-based NGS workflow (U2Bio, Bangkok, Thailand). Obtained sequence were analyzed and compared with reference *bla*_NDM_ variants available in GenBank database *via* the National Center for Biotechnology Information (NCBI). The complete *bla*_NDM_ open reading frames (ORF) were analyzed by translating the nucleotide sequences and comparing the corresponding full-length amino acid sequences using NCBI ORF finder. Allele designation was based on concordance of the complete ORF at both the nucleotide and protein sequence levels.

### Determination of antibiotic susceptibility patterns

Antimicrobial susceptibility profiles of all *bla*_NDM_ positive *A. baumannii* isolates were determined using the Kirby–Bauer disc diffusion method. Results were interpreted in accordance with the Clinical and Laboratory Standards Institute (CLSI) guidelines (CLSI, 2023). Twelve antimicrobial agents were evaluated; piperacillin–tazobactam (100/10 µg), ceftazidime (30 µg), cefepime (30 µg), cefotaxime (30 µg), ceftriaxone (30 µg), imipenem (10 µg), meropenem (10 µg), gentamicin (10 µg), amikacin (30 µg), tetracycline (30 µg), ciprofloxacin (5 µg), and trimethoprim–sulfamethoxazole (1.25/23.75 µg). Plates were incubated at 37 °C for 24 h, after which inhibition zone diameters were measured and interpreted as susceptible, intermediate, or resistant. Multidrug resistant *A. baumannii* (MDRAB), and extensively drug-resistant *A. baumannii* (XDRAB) were classified according to the criteria proposed by Magiorakos et al. (2012). The minimum inhibitory concentration (MIC) of colistin was determined in accordance with CLSI recommendations (CLSI, 2023). Briefly, two-fold serial dilutions of colistin were prepared in cation-adjusted Mueller–Hinton broth (CA-MHB) to obtain final concentrations ranging from 1 to 32 µg/mL. An equal volume of standardized bacterial suspension was added to each well to achieve a final volume of 100 µL per well. *A. baumannii* ATCC 19606 was used as the quality control strain for the disk diffusion assay, whereas *Pseudomonas aeruginosa* ATCC 27853 was used for colistin MIC determination.

### Whole genome sequencing

Four representative *bla*_NDM_-positive *A. baumannii* isolates (AB120, CR23, CR67-18, and CR67-72) were selected for whole-genome sequencing (WGS) based on their distinct combinations of *bla*_NDM_ variant (*bla*_NDM-1_ and *bla*_NDM-5_), antimicrobial resistance genes profile and years of isolation (2014, 2018, 2024). This selection strategy was intended to capture the genomic diversity represented among the available *bla*_NDM_-positive isolates. To compare the genomic context, WGS data from previous study of four additional *bla*_NDM_ positive isolates from another hospital were included (Kitti et al., 2023). These consisted of one newly sequenced isolate (AC48) and three publicly available isolates (AC02, AC09, and AC23) retrieved from the NCBI Sequence Read Archive (SRA) under BioProject PRJNA862456. Genomic DNA was extracted using PureDirex Genomics DNA Isolation Kit (BIO-HELIX, New Taipei City, Taiwan). DNA concentration and purity were assessed using a nanodrop spectrophotometer. Sequencing libraries were prepared and sequenced on Illumina HiSeq 2500-PE125 platform at Macrogen (Seoul, South Korea). The sequencing data for all eight *A. baumannii* isolates have been deposited in NCBI’s database under Sequence Read Archive (SRA) with Bio project PRJNA1426746

### Genome assembly and bioinformatics analysis

Quality control and adapter trimming of raw reads were performed to quality assessment using Trim Galore v0.6.7, followed by de novo assembly with Unicycler v0.4.8 using default parameters (Wick et al., 2017). Following assembly, only contigs longer than 300 bp were retained for downstream analyses. Pangenome analysis of the eight *A. baumannii* genomes was conducted using the anvi’o (version 9) pangenomic workflow (Delmont & Eren, 2018; Eren et al., 2021). A single-nucleotide polymorphism (SNPs) phylogenetic tree was reconstructed using CSI Phylogeny (Kaas et al., 2014). This tree was visualized and edited using Interactive Tree of Life (iTOL) (https://itol.embl.de/). Genome typing using both the Oxford and Pasteur multilocus sequence typing (MLST) schemes, together with prediction of capsule (K) and outer core lipooligosaccharide (OC) loci, was performed using Pathogenwatch. To predict whether assembled contigs originated from plasmids or chromosomes, Platon (Version 1.8) was employed through the oschwengers GitHub implementation (Schwengers et al., 2021). To identify *bla*_NDM_-carrying contigs, assembled sequences were screened against a local Comprehensive Antibiotic Resistance Database (CARD) using BLASTn with thresholds of ≥95% nucleotide identity and ≥90% query coverage. Approximately 10-kb flanking regions surrounding *bla*_NDM_ were extracted from representative contigs and annotated using Prokka v1.14.6 (Seemann, 2014). Gene synteny and sequence homology were visualized using GenoFig v1.1 (Branger & Leclercq, 2024).

## Results

### Prevalence of *A. baumannii* isolates

The prevalence of *bla*_NDM_-positive *A. baumannii* among the collected isolates was 19.23% (55/286). Based on hospital ward distribution, most isolates were recovered from intensive care units (ICUs) (20/55, 36.36%), followed by medical wards (19/55, 34.55%) and surgical wards (16/55, 29.09%). Most isolates were obtained from sputum specimens (49/55, 89.09%), whereas blood and urine specimens each accounted for 3.64% (2/55). Pus and other body fluid specimens each represented 1.82% (1/55).

### Antibiotic susceptibility profile of *bla*_NDM_ positive *A. baumannii* isolates

All *bla*_NDM_ positive *A. baumannii* isolates were subjected to antimicrobial susceptibility testing. Susceptibility to colistin was determined separately using the broth microdilution method to determine minimum inhibitory concentrations (MICs). The antimicrobial susceptibility profiles are shown in Fig. 1A and Table S2. All isolates were resistant to ceftriaxone, cefepime, meropenem, and imipenem (100% each). High resistance rates were also observed for cefotaxime (98.18%), ceftazidime (98.18%), and piperacillin–tazobactam (94.55%). Resistance to ciprofloxacin, gentamicin, amikacin, tetracycline, and trimethoprim–sulfamethoxazole was detected in 83.64%, 81.82%, 70.91%, 69.09%, and 32.73%, respectively. Notably, broth microdilution assay identified 36.36% (20/55) of isolates as colistin-resistant, with MIC values ranging from four to eight µg/mL. The prevalence of CRAB, MDRAB, and XDRAB among the 55 *bla*_NDM_ positive isolates was 100% (55/55), 90.91% (50/55), and 18.18% (10/55), respectively (Fig. 1B and Table S2). Based on the applied classification criteria, all XDRAB isolates were encompassed within the MDRAB category.

**Figure 1:**
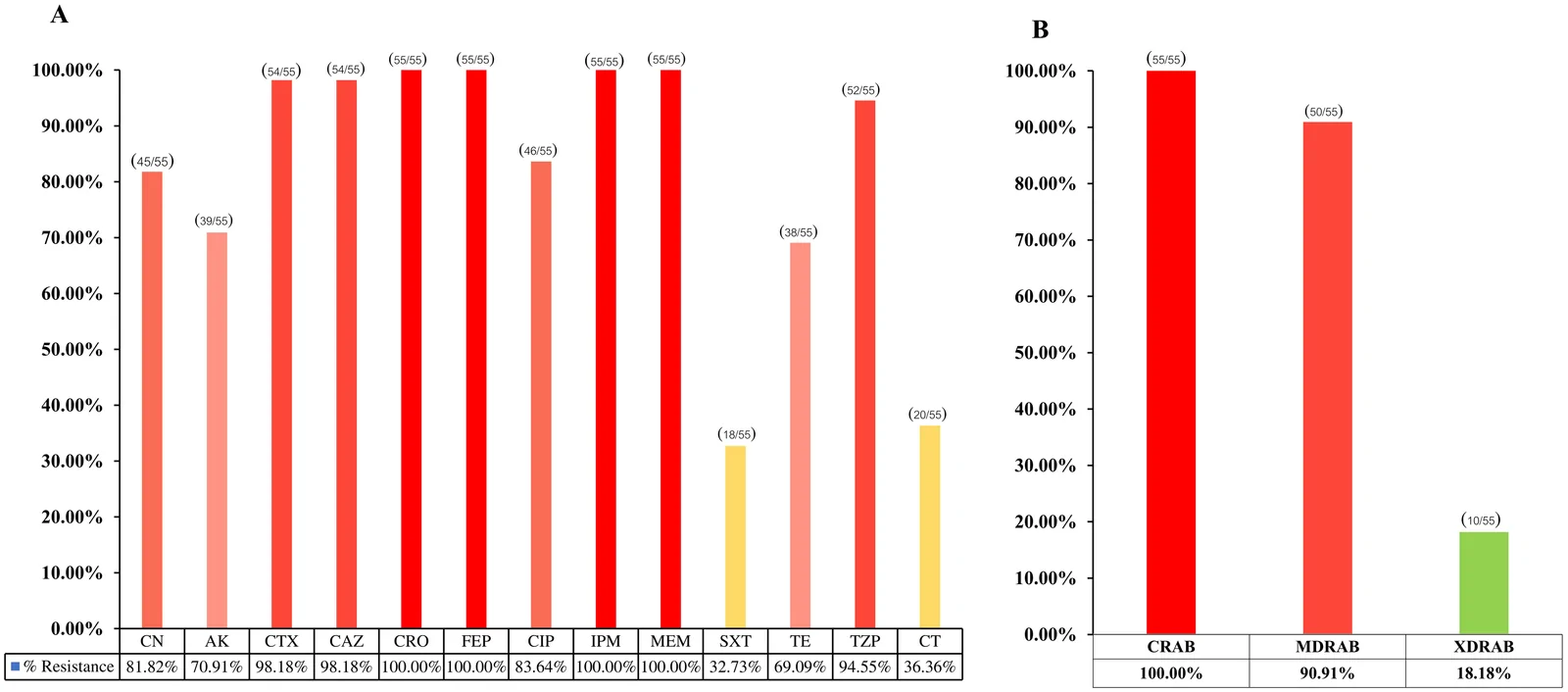
Antimicrobial resistance profiles of *bla*_NDM_-positive *Acinetobacter baumannii* isolates. (A) Percentage of isolates resistant to individual antimicrobial agents. gentamicin (CN), amikacin (AK), cefotaxime (CTX), ceftazidime (CAZ), ceftriaxone (CRO), cefepime (FEP), ciprofloxacin (CIP), imipenem (IPM), meropenem (MEM), trimethoprim/sulfamethoxazole (SXT), tetracycline (TE), piperacillin/tazobactam (TZP), and colistin (CT). (B) Distribution of carbapenem-resistant (CRAB), multidrug-resistant (MDRAB), and extensively drug-resistant (XDRAB).

### Antibiotic resistance genes, IS*Aba1*, IS*Aba125*, and characterization of *bla*_NDM_ genes

The molecular profiles of antimicrobial resistance genes detected among the *A. baumannii* isolates are shown in Fig. 2. The intrinsic *bla*_OXA-51_ was detected in all isolates, whereas *bla*_OXA-24_ was not identified in any isolates. The prevalence of *bla*_OXA-23_ and *bla*_OXA-58_ in the isolates was 85.45% (47/55) and 16.36% (9/55), respectively (Table S3). Sequence analysis of the *bla*_NDM_ gene demonstrated that 49 isolates (89.09%) shared 97%–100% nucleotide identity with *bla*_NDM-1_, whereas six isolates (10.91%) exhibited 100% identity with *bla*_NDM-5_ (Table S4). The *bla*_NDM-1_-positive *A. baumannii* isolates were identified throughout the study period, whereas *bla*_NDM-5_-positive isolates were detected exclusively among isolates collected in 2024.

**Figure 2:**
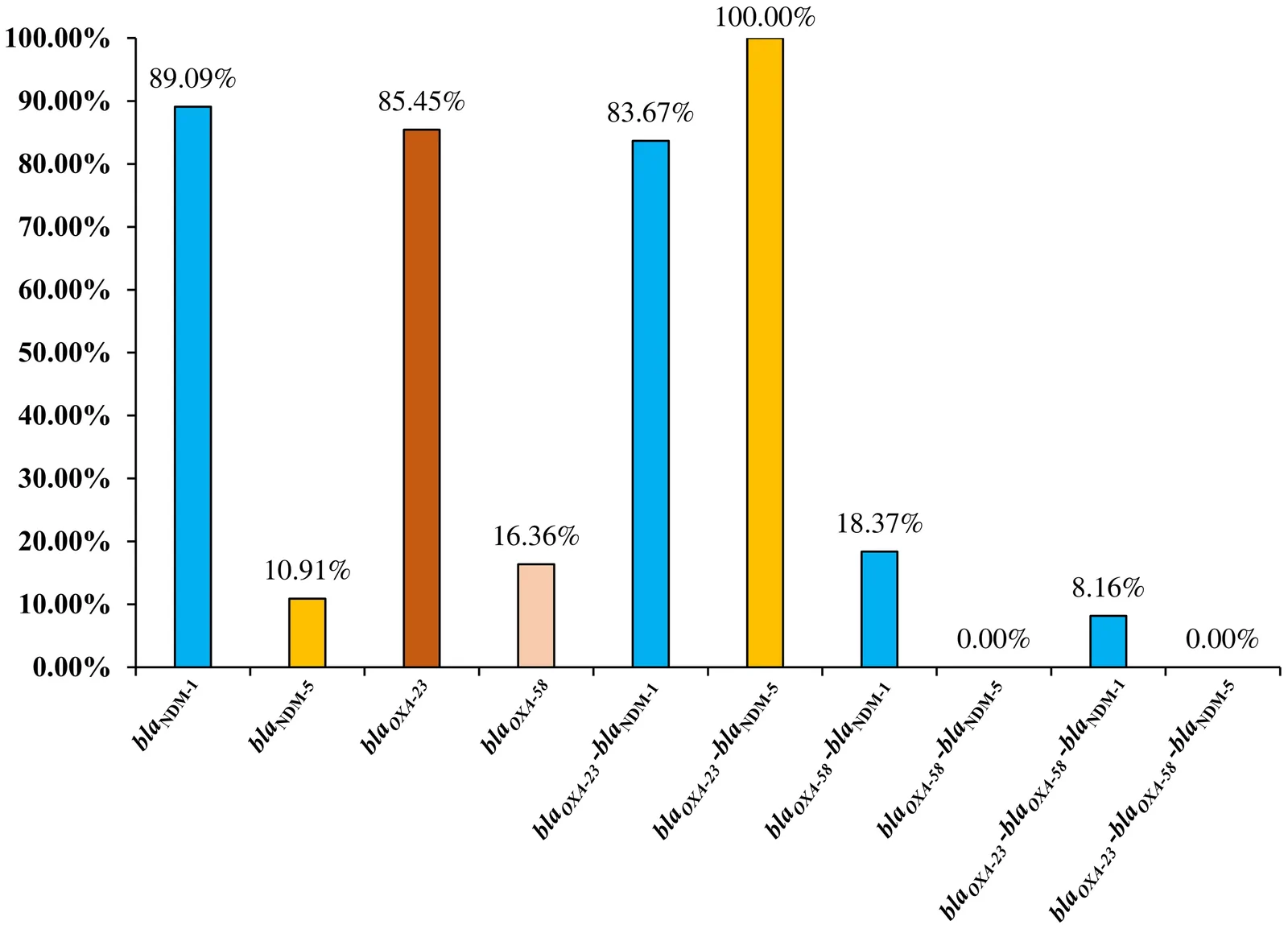
Dissemination of *bla*_NDM-1_, *bla*_NDM-5_, *bla*_OXA-23_, *bla*_OXA-58_ and co-occurrence of resistance genes of *bla*_NDM_ positive *A. baumannii*.

The co-occurrence of resistance genes was frequently observed. The most common combination was *bla*_OXA-23_-*bla*_NDM- 1_ detected in 83.67% (41/49) of *bla*_NDM-1_ positive isolates. Other combinations included *bla*_OXA-58_-*bla*_NDM-1_ (18.37%, 9/49) and *bla*_OXA-23_-*bla*_NDM- 5_(100%, 6/6). Additionally, the simultaneous presence of *bla*_OXA-23_-*bla*_OXA-58_-*bla*_NDM- 1_ was identified in 8.16% (4/49) of isolates. Notably, the co-occurrence of *bla*_OXA- 23_ and *bla*_NDM-1_ was consistently associated with the CRAB phenotype, which showed high resistance (100%) to carbapenems and cephalosporins (100%). Isolates carrying *bla*_OXA- 23_-*bla*_NDM-5_ demonstrated a comparable resistance profile. No distinct phenotypic differences were observed among the major genotype groups (Table S5). Among the insertion sequences examined, IS*Aba1* and IS*Aba125* were detected in 89.09% (49/55) and 81.82% (45/55) of *bla*_NDM_ positive *A. baumannii* isolates, respectively. Among the IS*Aba1* positive isolates, most harbored IS*Aba1* upstream of *bla*_OXA-23_.

**Table 1:** Genome features of *bla*_*NDM*_ positive isolates and their characteristic of *bla*_*NDM*_ contigs.

**Strains**		**Genome features** ^*^									***bla***_**NDM**_ ** contigs**			
	**Number of contig**	**number of reads**	**Genome size**	**Total length**	**N50**	**%GC**	**K locus**	**OC**	**MLST** **(oxford)**	**MLST** **(Pasteur)**	**Type**	**Contig ID**	**Contig size** (bp)	**Plasmid prediction** ^**^
AB120	49	7,322,151	3,933,324	3,930,720	228,140	38.97	KL8	OCL2	642	23	NDM-1	26	7,924	Plasmid
AC48	137	5,487,335	3,902,725	3,893,046	61,076	39	KL96	OCL13	145	213		77	7,925	Plasmid
CR23	56	3,728,457	4,101,545	4,096,442	201,426	39.1	KL24	OCL7	355	16		35	7,925	Plasmid
CR67-72	61	3,591,316	4,079,573	4,074,179	199,453	38.99	KL24	OCL7	355	16		38	7,925	Plasmid
AC02	79	15,095,548	4,016,797	4,014,312	167,724	38.9	KL3	OCL1	195	2	NDM-5	45	9,919	Plasmid
AC09	65	14,910,840	3,934,990	3,934,040	160,681	38.98	KL49	OCL1	1684	2		42	9,919	Plasmid
AC23	65	14,497,076	3,925,340	3,922,544	161,945	38.98	KL3	OCL1	195	2		43	9,919	Plasmid
CR67-18	59	7,481,988	4,007,766	4,006,027	130,859	38.86	KL16	OCL1	1684	2		41	9,919	Plasmid

**Notes.**

* Genome feature is derived from draft genome sequence data from illumina sequencing.

** Plasmid prediction was performed using Platon via oschwengers GitHub (github.com/oschwengers/platon).

**Figure 3:**
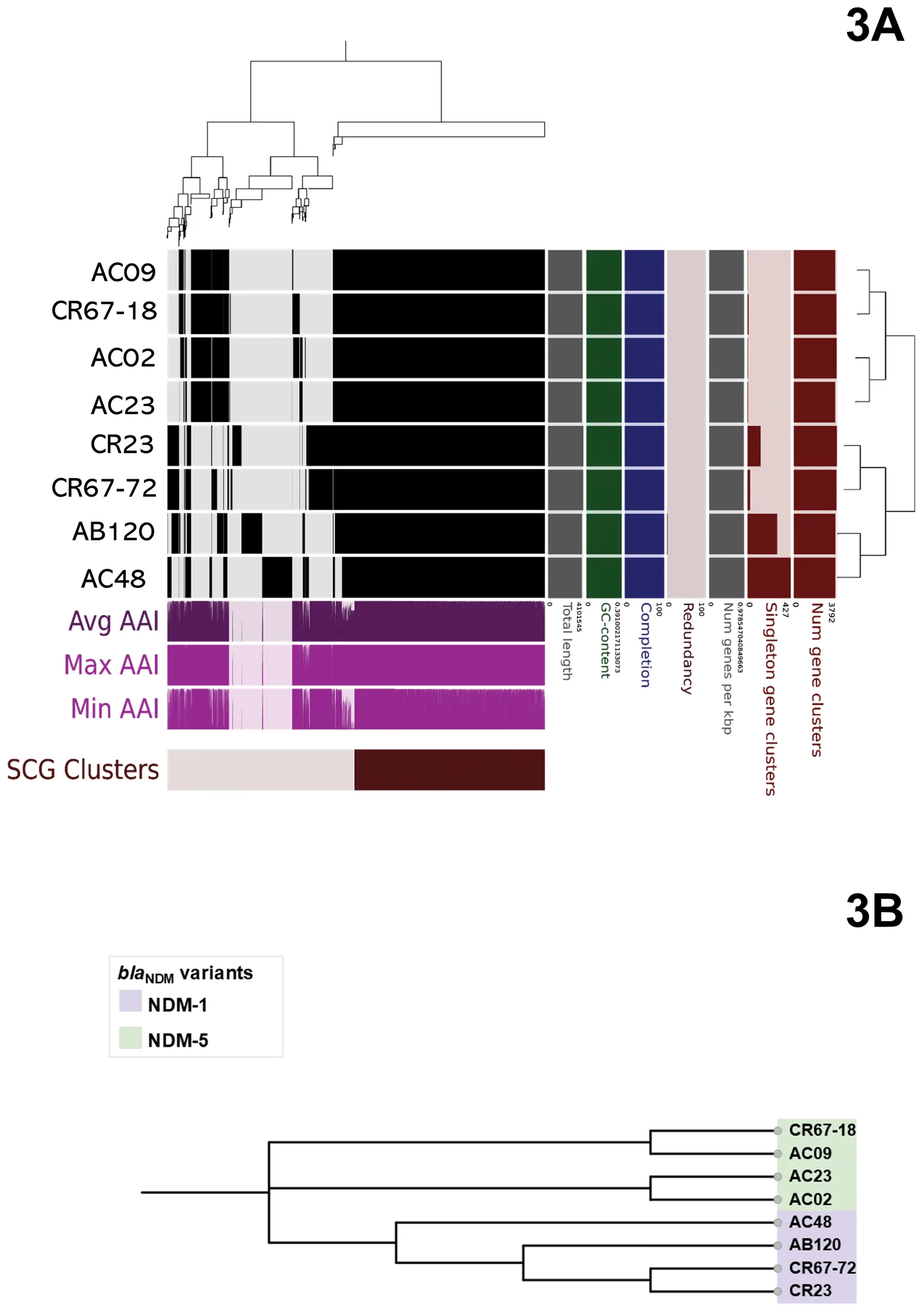
Pangenome analysis and Core-genome single-nucleotide polymorphism (SNP) phylogenetic tree of *Acinetobacter baumannii* isolates carrying *bla*_NDM-1_ and *bla*_NDM-5_. (A) Pangenome analysis and hierarchical clustering showed the presence or absence matrix of gene clusters generated from *bla*_NDM-_ positive *A. baumannii* genomes indicating a highly conserved core genome with modest accessory genome diversity. Black and gray cells represent the presence and absence of gene clusters, respectively. Hierarchical clustering based on gene content segregated isolates into *bla*_NDM-1_ (isolates (AB120, AC48, CR23, and CR67-72) and *bla*_NDM-5_ (AC02, AC09, AC23, and CR67-18) lineages. Genome statistics (genome length, GC content, completeness, redundancy, genes per kilobase, singleton gene clusters, and total gene clusters) are shown on the right. The lower panel is single-copy core gene (SCG) clusters. (B) Core-genome SNP phylogeny exhibits high congruence with the pangenome clustering. The isolates are distinctly separated into two clades perfectly matching their NDM variants: *bla*_NDM-1_ (shaded in purple) and *bla*_NDM-5_ lineage (shaded in green).

### Genomic context of *bla*_NDM-1_ and *bla*_NDM-5_ in *A. baumannii*

The draft genomes of four *bla*_NDM-1_-positive and four *bla*_NDM-5_-positive *A. baumannii* isolates exhibited variation in genome size, GC content, capsular polysaccharide (K-locus), and outer core lipooligosaccharide (OC-locus) types (Table 1). Multilocus sequence typing (MLST), based on the Pasteur scheme revealed that *bla*_NDM-1_-positive *A. baumannii* belonged to three sequence types (ST16, ST23, and ST213), whereas all *bla*_NDM-5_-positive *A. baumannii* belonged to ST2. Pangenome analysis of all eight *A. baumannii* isolates demonstrated a highly conserved core genome (Fig. 3A). Hierarchical clustering based on gene presence–absence profiles (Fig. 3A), together with SNP- based phylogenetic analysis (Fig. 3B), separated the isolates into two distinct clusters corresponding to the *bla*_NDM-1_ and *bla*_NDM-5_ lineages, respectively. Analysis of the genomic context surrounding *bla*_NDM_ revealed highly conserved genetic architectures among isolates carrying the same *bla*_NDM_ variant (Fig. 4). The four *bla*_NDM-1_-positive isolates (AB120, AC48, CR23, and CR67-72) displayed identical gene organization, with *bla*_NDM-1_ located downstream of the *ble* gene and followed by genes involved in cellular homeostasis (*trpF, dsbD*, and *cutA*) and chaperone function (*groS* and *groL*). Members of the IS91 family of insertion sequences were consistently detected upstream of this region. However, whole-genome sequencing did not identify IS*Aba*125 within the *bla*_NDM-1_-containing contigs (Fig. 4A). In contrast, the four *bla*_NDM-5_-positive isolates (AC02, AC09, AC23, and CR67-18) exhibited a distinct but highly conserved genetic organization. In these isolates, *bla*_NDM-5_ was consistently preceded by several antimicrobial resistance genes, including members of the *ant(3″)* gene family, encoding aminoglycoside 3*″*-adenylyltransferase, *emrE*, encoding a small multidrug resistance (SMR) efflux pump, f*olP*, encoding dihydropteroate synthase associated with sulfonamide resistance, and *ble*, which confers bleomycin resistance. Multiple MGEs, including IS91 family transposases, were identified upstream of *bla*_NDM-5,_ whereas IS*Aba*125 was consistently located downstream of the resistance gene (Fig. 4B). To investigate the genomic location of *bla*_NDM_, all *bla*_NDM_-containing contigs were analyzed using Platon. The analysis predicted that *bla*_NDM_-containing contigs from all eight *A. baumannii* isolates were plasmid associated (Table 1).

## Discussion

In the present study, most specimens were recovered from sputum (89.09%) obtained from hospitalized patients, indicating that respiratory tract infections were the predominant clinical presentation. This finding also suggests frequent exposure to invasive airway management, including mechanical ventilation. Furthermore, 36.36% of *A. baumannii* isolates were obtained from patients admitted to ICUs, highlighting the strong association between this pathogen and critically ill patients. Consistent with this finding, the World Health Organization classified *A. baumannii* as a critical-priority antimicrobial-resistant pathogen, which reflects its significant contribution to severe, difficult-to-treat infections and excess mortality among ICU patients (Vázquez-López et al., 2020).

**Figure 4:**
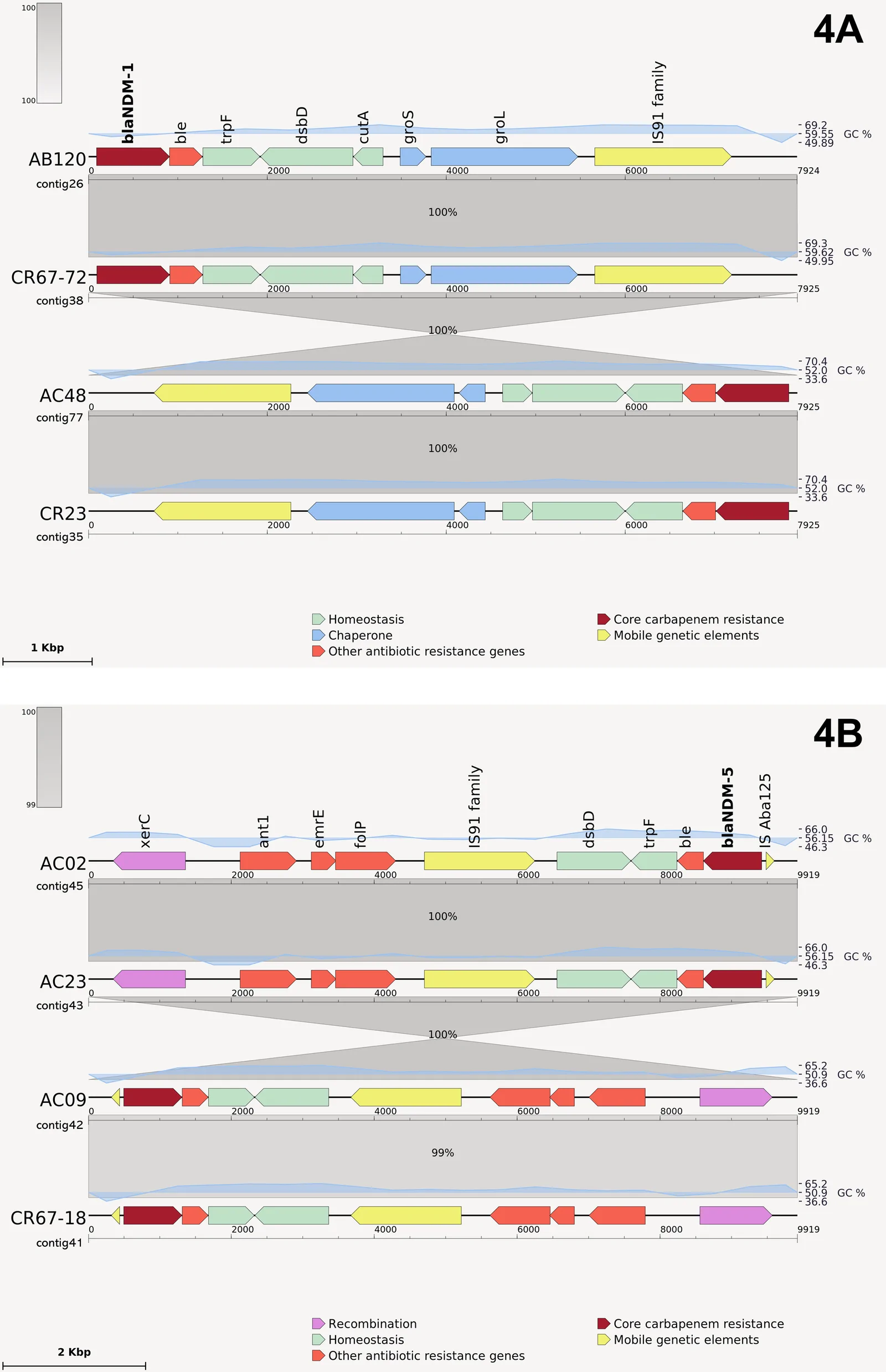
Genetic environments surrounding *bla*_NDM_ genes in *A. baumannii* isolates. (A) Genetic contexts and synteny comparison of four *bla*_NDM-1_ positive isolates (AB120, AC48, CR23, and CR67-72). The *bla*_NDM-1_ gene context was located within a highly conserved genomic region comprising the IS91 family transposase, *groL, groS, cutA, dsbD, trpF*, and *ble* genes. The genomic organization was identical among all four isolates, exhibiting 100% sequence identity across the aligned region. (B) Genetic contexts and synteny comparison of four *bla*_NDM-5_ positive isolates (AC02, AC09, AC23, and CR67-18). The *bla*_NDM-5_ gene context showed a highly conserved gene organization consisting of *xerC, ant1, emrE, folP*, an IS91 family transposase, *dsbD, trpF*, ble, and a downstream IS*Aba*125 insertion sequence. Arrows represent open reading frames and indicate the direction of transcription. Genes are color-coded according to their predicted functions: recombination (purple), homeostasis (light green), chaperone (blue), other antibiotic resistance genes (orange), *bla*_NDM_ (red), and mobile genetic elements (yellow). Shaded regions between sequences indicate nucleotide similarity, with percentage identity labeled. The scale bar corresponds to 2 kbp.

High levels of resistance to both cephalosporins and carbapenems were observed among the *A. baumannii* isolates. In this study, nearly all isolates exhibited resistance to carbapenems, with resistance rates of 100% for meropenem and imipenem. Notably, colistin, which is often considered as a last-resort therapeutic option for multidrug-resistant *Acinetobacter* infections, showed a resistance rate of 36.36%, raising serious concerns regarding the diminishing effectiveness of one of the few remaining therapeutic options. These resistance patterns are consistent with previous reports from Thailand, indicating the sustained and widespread circulation of highly drug-resistant *A. baumannii* in clinical settings (Thirapanmethee et al., 2020). These findings emphasize the urgency for the rational use of antibiotics, particularly carbapenems and colistin, to effectively manage and mitigate the spread of antibiotic resistance.

An exceptionally high prevalence of CRAB was observed (100%), with 90.91% of isolates classified as MDRAB and 18.18% as XDRAB. Comparable CRAB prevalence rates have been reported in previous studies from Thailand, indicating widespread dissemination and sustained endemicity of carbapenem-resistant strains in clinical settings (Thirapanmethee et al., 2020; Santajit et al., 2023). CRAB infections are frequently associated with outbreaks in ICUs, where the organism poses substantial challenges to infection prevention and control challenges due to their ability to survive for prolonged periods on abiotic surfaces and within the hospital environment (Byun et al., 2021). Despite the escalating prevalence of resistance, carbapenems continue to be widely prescribed for the treatment of severe *Acinetobacter* infections, thereby maintaining selective pressure that may facilitate the emergence and persistence of highly resistant clones.

The production of NDM enzymes plays a pivotal role in mediating high-level carbapenem resistance in *A. baumannii*. In this study, *bla*_NDM-1_ was the dominant NDM variant among *A. baumannii* isolates, accounting for 89.09% of *bla*_NDM_-positive strains. Previous studies from Thailand have consistently reported a high prevalence of *bla*_NDM-1_ among *A. baumannii* isolates collected from hospitals in both northern and southern regions, thereby supporting the extensive circulation of NDM-producing strains nationwide (Leungtongkam et al., 2018; Chukamnerd et al., 2022). These findings are also consistent with international reports documenting the global distribution of NDM-producing *A. baumannii* across Asia, Europe, and the Americas, highlighting the global dissemination and clinical relevance of this resistance determinant (Castanheira, Mendes & Gales, 2023). Additionally, the identification of *bla*_NDM-5_ in 10.91% of the isolates is of notable epidemiological importance. Although *bla*_NDM-5_ has been more commonly reported in *Enterobacterales*, its identification in *A. baumannii* suggests potential interspecies horizontal gene transfer events and highlights the expanding genetic diversity of MBLs within this pathogen. A previous study from Thailand reported the first identification of NDM-5-producing *A. baumannii* from both clinical and environmental samples, underscoring the environmental persistence and transmission potential of this resistance determinant (Kitti et al., 2023). Similarly, studies from India have consistently reported the presence of *bla*_NDM-5_ among *A. baumannii* isolates, suggesting its increasing establishment in specific geographic regions (Khalid et al., 2020).

This study provides genomic evidence that carbapenem resistance mediated by *bla*_NDM-1_ and *bla*_NDM-5_ in *A. baumannii* collected from two hospitals in Thailand is associated with highly conserved mobile resistance cassettes. Comparative analysis of the genomic regions surrounding *bla*_NDM_ in the eight sequenced isolates revealed highly conserved gene organization with remarkable synteny among isolates carrying the same *bla*_NDM_ variant. The *bla*_NDM-1_ gene context consisted of an IS*91* family transposase followed by the *groL, groS, cutA, dsbD, trpF*, and *ble* genes. In contrast, the *bla*_NDM-5_ genetic context comprised *xerC, ant1, emrE, folP*, an IS*91* family transposase, *dsbD, trpF*, *ble, bla*_NDM-5_, and a downstream IS*Aba125* insertion sequence. Notably, the conserved *dsbD, trpF*, and *ble* gene cluster identified in all *bla*_NDM_-positive isolates has previously been reported as part of conjugative IncA/C2 plasmid carrying *bla*_NDM_ in *K. pneumoniae* (Zhao et al., 2022). The observation of this genetic module suggests that the *bla*_NDM_ loci identified in the present study may have originated from plasmid-associated elements. In addition, the WGS data were analyzed using Platon, a bioinformatics tool designed to identify and characterize plasmid-associated contigs in bacterial draft genome generated from short-read sequencing. Platon predicted that both *bla*_NDM-1_ and *bla*_NDM-5_ containing contigs from this study were plasmid associated. This finding is consistent with a recent study from Brazil reporting plasmid localization of *bla*_NDM-1_ gene (Rodrigues et al., 2024).

Pangenome analysis and SNPs phylogenetic tree separated the eight sequenced isolates into two distinct clusters corresponding to the *bla*_NDM-1_ and *bla*_NDM-5_ genotypes. Notably, these two groups differed in capsular polysaccharide (K-locus) type, OC-locus types suggesting that *bla*_NDM-1_ and *bla*_NDM-5_ are maintained within distinct evolutionary lineages of *A. baumannii*. Furthermore, the distinct genomic groupings suggests that the acquisition and dissemination of *bla*_NDM-1_ and *bla*_NDM-5_ likely occurred through independent evolutionary events and may have been driven by different transmission dynamics. MLST analysis revealed that the four *bla*_NDM-1_-positive isolates belonged to three distinct sequence types (ST16, ST23, and ST213), despite carrying *bla*_NDM-1-_associated genetic cassettes with highly conserved genomic context. The present of identical genetic arrangement across genetically diverse sequence types is supports the hypothesis of plasmid mediated horizontal acquisition of the *bla*_NDM-1_-associated cassette. In contrast, the predominance of ST2 among three of four *bla*_NDM-5_-positive isolates, which originated from a previously reported study, raises the possibility that this isolate represents a previously described outbreak or clonal expansion.

The genetic architectures identified in this study closely resemble NDM-associated platforms reported worldwide, where *bla*_NDM_ is frequently associated with IS*Aba*125, Tn125 composite transposons, class 1 integrons, and various insertion sequences (such as IS26 and ISCR elements) that facilitate mobilization and stabilization in the chromosome or plasmid-derived fragments (Acman et al., 2022). Interestingly, polymerase chain reaction (PCR) analysis demonstrated that all *bla*_NDM_ positive isolates carried IS*Aba125,* whereas short-read Illumina genome assemblies identified IS*Aba125* only downstream of the *bla*_NDM-5_ locus and failed to assemble IS*Aba125* within the *bla*_NDM-1_-harboring contigs. This discrepancy most likely reflects the inherent limitations of short-read sequencing in resolving repetitive insertion sequences and complex mobile genetic elements. Consequently, IS*Aba125* was likely located elsewhere in the genome, although its precise genomic position relative to *bla*_NDM-1_ could not be determined. Therefore, the direct role of IS*Aba125* in the mobilization or transcriptional regulation of *bla*_NDM-1_ remains unresolved and warrants further investigation using complete genome assemblies generated with long-read sequencing technologies.

In contrast, IS*91* family insertion sequences were consistently identified upstream of both *bla*_NDM-1_ and *bla*_NDM-5_. The presence of IS*91*-like elements in close proximity to *bla*_NDM_ is noteworthy because these elements may facilitate the mobilization and dissemination of carbapenem resistance genes through transposition between chromosome and plasmid DNA.

Associations between *bla*_NDM_ and IS*91*-like elements have been reported in several clinically important Gram-negative pathogens, including *Escherichia coli* and *Pseudomonas aeruginosa* (Tang et al., 2025; Wang et al., 2026). Therefore, our findings are consistent with previous genomic studies that demonstrated IS-mediated transposition’s role as an important mechanism driving the dissemination of carbapenemase genes among successful hospital-adapted *A. baumannii* lineages (Schultz et al., 2016).

In conclusion, this study characterizes the prevalence, antimicrobial resistance profiles, and genomic features of *bla*_NDM_-positive *A. baumannii* isolates collected from a tertiary-care hospital in northern Thailand. The isolates demonstrated extensive resistance to carbapenems and cephalosporins and harbored *bla*_NDM-1_ and *bla*_NDM-5_ genes. Comparative genomic analysis of *bla*_NDM-1_ and *bla*_NDM-5_-associated region from two hospitals in Thailand revealed highly conserved *bla*_NDM_ genetic context surrounding the *bla*_NDM_ genes, including conserved association with mobile genetic elements. These finding enhance our understanding of the molecular epidemiology and evolutionary dynamic of *bla*_NDM_ in *A. baumannii* and provide genomic evidence to inform antimicrobial resistance surveillance, strengthen infection prevention and control measures, and guide strategies aimed at limiting the dissemination of MDRAB and CRAB in Thailand.

## Supplemental Information

10.7717/peerj.21848/supp-1 Supplemental Information 1 Clinical isolates of Acinetobacter baumanni use for identifing of blaNDM positive

10.7717/peerj.21848/supp-2 Supplemental Information 2 Supplemental Tables

10.7717/peerj.21848/supp-3 Supplemental Information 3 Raw data from Platon analysis

## Additional Information and Declarations

### Data Availability

The following information was supplied regarding data availability: The raw measurements are available in the Supplemental Files. The raw sequence data are available at NCBI BioProject: PRJNA1426746.
