# Supplementary material for: Conserved mobile genetic contexts associated with blaNDM-1 and blaNDM-5in Acinetobacter baumannii isolated from two hospitals in Thailand
Source: PeerJ. 2026 Oct 27;14:e21848. doi: 10.7717/peerj.21848 (PMC13721886; doi:10.7717/peerj.21848)
Supplement: Supplementary file 1 [file peerj-14-21848-s002.docx]

**Table S1. List of Primers used in this study.**

| **Target genes** | **Primer name** | **Sequence** (**5’-3’**) | **Amplicon size (bp)** | **Annealing temp.** | **References** |
| --- | --- | --- | --- | --- | --- |
|  |  |  |  | **(^o^C)** |  |
| *16S rRNA* | 16s rRNA F | AGAGTTTGATCCTGGCTCAG | 1,500 | 58 | Misbah S, Hassan H, Yusof MY, Hanifah YA, AbuBakar S. Genomic species identification of Acinetobacter of clinical isolates by 16S rDNA sequencing. Singapore Med J. 2005; 46: 461–464. PMID:16123830. |
|  | 16s rRNA R | ACGGCTACCTTGTTACGACTT |  |  |  |
| *rpo*B | rpoB F | CCTTCATGACCTGGAAYGGNTA | 940 | 59 | Wang J, Ruan Z, Feng Y, Fu Y, Jiang Y, Wang H, et al. Species distribution of clinical Acinetobacter isolates revealed by different identification techniques. PLoS One. 2014; 9:e104882. https://doi.org/10. 1371/journal.pone.0104882 PMID: 25120020. |
|  | rpoB R | TCCAGGATCTGNCCNACRTTCAT |  |  |  |
| *bla*_OXA-51_ | blaOXA-51 F | TAATGCTTTGATCGGCCTTG | 353 | 52 | Woodford N, Ellington MJ, Coelho JM, Turton JF, Ward ME, Brown S, et al. Multiplex PCR for genes encoding prevalent OXA carbapenemases in Acinetobacter spp. Int J Antimicrob Agents. 2006; 27: 351–353. https://doi.org/10.1016/j.ijantimicag.2006.01.004 PMID: 16564159. |
|  | blaOXA-51 R | TGGATTGCACTTCATCTTGG |  |  |  |
| *bla*_OXA-23_ | blaOXA-23 F | GATCGGATTGGAGAACCAGA | 501 | 52 | Woodford N, Ellington MJ, Coelho JM, Turton JF, Ward ME, Brown S, et al. Multiplex PCR for genes encoding prevalent OXA carbapenemases in Acinetobacter spp. Int J Antimicrob Agents. 2006; 27: 351–353. https://doi.org/10.1016/j.ijantimicag.2006.01.004 PMID: 16564159. |
|  | blaOXA-23 R | ATTTCTGACCGCATTTCCAT |  |  |  |
| *bla*_OXA-24_ | blaOXA-24 F | GGTTAGTTGGCCCCCTTAAA | 246 | 52 | Woodford N, Ellington MJ, Coelho JM, Turton JF, Ward ME, Brown S, et al. Multiplex PCR for genes encoding prevalent OXA carbapenemases in Acinetobacter spp. Int J Antimicrob Agents. 2006; 27: 351–353. https://doi.org/10.1016/j.ijantimicag.2006.01.004 PMID: 16564159. |
|  | blaOXA-24 R | AGTTGAGCGAAAAGGGGATT |  |  |  |
| *bla*_OXA-58_ | blaOXA-58 F | AAGTATTGGGGCTTGTGCTG | 599 | 52 | Woodford N, Ellington MJ, Coelho JM, Turton JF, Ward ME, Brown S, et al. Multiplex PCR for genes encoding prevalent OXA carbapenemases in Acinetobacter spp. Int J Antimicrob Agents. 2006; 27: 351–353. https://doi.org/10.1016/j.ijantimicag.2006.01.004 PMID: 16564159. |
|  | blaOXA-58 R | CCCCTCTGCGCTCTACATAC |  |  |  |
| *bla*_NDM_ | blaNDM- F | GGTTTGGCGATCTGGTTTTC | 621 | 52 | Poirel L, Walsh TR, Cuvillier V, Nordmann P. Multiplex PCR for detection of acquired carbapenemase genes. Diagn Microbiol Infect Dis. 2011; 70: 119–123. https://doi.org/10.1016/j.diagmicrobio.2010.12. 002 PMID: 21398074 |
|  | blaNDM- R | CGGAATGGCTCATCACGATC |  |  |  |
| *ISAba125* | ISAba125-F | TGTTGAAGCGATCCGTTGTT | 755 | 57 | Leungtongkam U, Thummeepak R, Wongprachan S, et al. Dissemination of blaOXA-23, blaOXA-24, blaOXA-58, and blaNDM-1 Genes of Acinetobacter baumannii Isolates from Four Tertiary Hospitals in Thailand. Microbial Drug Resistance. 2017;24(1):55-62. doi:10.1089/mdr.2016.0248 |
|  | ISAba125-R | GTGCGACAGTTTCAAAAGCCA |  |  |  |

**Table S2 Antibiotic susceptibility profile of blaNDM positive *A. baumannii* isolates**

|  | **Disc susceptibility test result** | | | | | | | | | | | | | colistin | MIC colisti µg/mL | **XDR-AB** | **MDR-AB** | **CR-AB** |
| --- | --- | --- | --- | --- | --- | --- | --- | --- | --- | --- | --- | --- | --- | --- | --- | --- | --- | --- |
|  | **CN** | **AK** | **CTX** | **CAZ** | **CRO** | **FEP** | **CIP** | **IPM** | **MEM** | **SXT** | **TE** | **TZP** | **CT** | resistance |  |  |  |  |
| 1 | R | S | R | R | R | R | R | R | R | R | S | R | 0 | No | 2 | Absent | Present | Present |
| 2 | R | S | R | R | R | R | R | R | R | R | R | R | 1 | Yes | 4 | Present | Absent | Present |
| 3 | R | S | R | R | R | R | R | R | R | R | S | R | 0 | No | 2 | Absent | Present | Present |
| 4 | S | S | R | R | R | R | R | R | R | S | S | S | 0 | No | 2 | Absent | Absent | Present |
| 5 | S | S | S | S | R | R | R | R | R | S | S | R | 1 | Yes | 4 | Absent | Present | Present |
| 6 | S | S | R | R | R | R | R | R | R | S | S | S | 0 | No | 2 | Absent | Absent | Present |
| 7 | S | S | R | R | R | R | R | R | R | S | S | S | 0 | No | 2 | Absent | Absent | Present |
| 8 | R | R | R | R | R | R | S | R | R | S | R | R | 1 | Yes | 4 | Absent | Present | Present |
| 9 | R | R | R | R | R | R | S | R | R | S | R | R | 0 | No | 2 | Absent | Present | Present |
| 10 | R | R | R | R | R | R | S | R | R | S | R | R | 1 | Yes | 4 | Absent | Present | Present |
| 11 | R | R | R | R | R | R | R | R | R | S | R | R | 1 | Yes | 4 | Absent | Present | Present |
| 12 | R | R | R | R | R | R | R | R | R | S | R | R | 1 | Yes | 4 | Absent | Present | Present |
| 13 | R | R | R | R | R | R | R | R | R | S | S | R | 0 | No | 2 | Absent | Present | Present |
| 14 | R | R | R | R | R | R | R | R | R | S | R | R | 1 | Yes | 4 | Absent | Present | Present |
| 15 | R | R | R | R | R | R | R | R | R | S | R | R | 1 | Yes | 4 | Absent | Present | Present |
| 16 | R | R | R | R | R | R | R | R | R | R | R | R | 0 | No | 2 | Present | Absent | Present |
| 17 | R | R | R | R | R | R | R | R | R | S | R | R | 1 | Yes | 4 | Absent | Present | Present |
| 18 | R | R | R | R | R | R | R | R | R | S | R | R | 1 | Yes | 4 | Absent | Present | Present |
| 19 | R | S | R | R | R | R | R | R | R | S | S | R | 0 | No | 2 | Absent | Present | Present |
| 20 | R | R | R | R | R | R | S | R | R | S | R | R | 1 | Yes | 4 | Absent | Present | Present |
| 21 | R | R | R | R | R | R | R | R | R | S | R | R | 0 | No | 2 | Absent | Present | Present |
| 22 | R | R | R | R | R | R | R | R | R | S | R | R | 0 | No | 2 | Absent | Present | Present |
| 23 | R | R | R | R | R | R | S | R | R | S | R | R | 0 | No | 2 | Absent | Present | Present |
| 24 | R | R | R | R | R | R | S | R | R | S | R | R | 0 | No | 2 | Absent | Present | Present |
| 25 | S | S | R | R | R | R | S | R | R | S | R | R | 0 | No | 2 | Absent | Present | Present |
| 26 | R | R | R | R | R | R | R | R | R | S | R | R | 0 | No | 2 | Absent | Present | Present |
| 27 | R | R | R | R | R | R | R | R | R | S | R | R | 0 | No | 2 | Absent | Present | Present |
| 28 | R | S | R | R | R | R | R | R | R | R | S | R | 0 | No | 2 | Absent | Present | Present |
| 29 | S | S | R | R | R | R | R | R | R | S | R | R | 0 | No | 2 | Absent | Present | Present |
| 30 | R | S | R | R | R | R | R | R | R | R | S | R | 0 | No | 2 | Absent | Present | Present |
| 31 | R | R | R | R | R | R | R | R | R | S | R | R | 0 | No | 2 | Absent | Present | Present |
| 32 | R | R | R | R | R | R | R | R | R | S | R | R | 0 | No | 2 | Absent | Present | Present |
| 33 | S | S | R | R | R | R | R | R | R | S | R | R | 0 | No | 2 | Absent | Present | Present |
| 34 | R | R | R | R | R | R | R | R | R | S | R | R | 1 | Yes | 4 | Absent | Present | Present |
| 35 | R | R | R | R | R | R | S | R | R | S | R | R | 1 | Yes | 4 | Absent | Present | Present |
| 36 | R | R | R | R | R | R | R | R | R | S | R | R | 1 | Yes | 4 | Absent | Present | Present |
| 37 | R | R | R | R | R | R | R | R | R | S | R | R | 1 | Yes | 4 | Absent | Present | Present |
| 38 | R | R | R | R | R | R | R | R | R | S | R | R | 1 | No | 4 | Absent | Present | Present |
| 39 | R | R | R | R | R | R | R | R | R | R | S | R | 0 | No | 2 | Absent | Present | Present |
| 40 | R | R | R | R | R | R | R | R | R | R | S | R | 0 | No | 2 | Absent | Present | Present |
| 41 | R | R | R | R | R | R | R | R | R | R | S | R | 1 | Yes | 4 | Absent | Present | Present |
| 42 | R | R | R | R | R | R | R | R | R | R | S | R | 1 | Yes | 4 | Absent | Present | Present |
| 43 | S | S | R | R | R | R | R | R | R | S | S | R | 0 | No | 2 | Absent | Present | Present |
| 44 | S | S | R | R | R | R | R | R | R | S | S | R | 1 | Yes | 8 | Absent | Present | Present |
| 45 | R | R | R | R | R | R | R | R | R | S | R | R | 0 | No | 2 | Absent | Present | Present |
| 46 | S | S | R | R | R | R | R | R | R | S | S | R | 0 | No | 2 | Absent | Present | Present |
| 47 | R | R | R | R | R | R | S | R | R | S | R | R | 1 | Yes | 4 | Absent | Present | Present |
| 48 | R | R | R | R | R | R | R | R | R | R | R | R | 0 | No | 1 | Present | Present | Present |
| 49 | R | R | R | R | R | R | R | R | R | R | R | R | 0 | No | 1 | Present | Present | Present |
| 50 | R | R | R | R | R | R | R | R | R | R | R | R | 0 | No | 1 | Present | Present | Present |
| 51 | R | R | R | R | R | R | R | R | R | R | R | R | 0 | No | 2 | Present | Present | Present |
| 52 | R | R | R | R | R | R | R | R | R | R | R | R | 0 | No | 2 | Present | Present | Present |
| 53 | R | R | R | R | R | R | R | R | R | R | R | R | 0 | No | 2 | Present | Present | Present |
| 54 | R | R | R | R | R | R | R | R | R | R | R | R | 0 | No | 2 | Present | Present | Present |
| 55 | R | R | R | R | R | R | R | R | R | R | R | R | 0 | No | 2 | Present | Present | Present |
|  | **45** | **39** | **54** | **54** | **55** | **55** | **46** | **55** | **55** | **18** | **38** | **52** | **20** | **20** |  | **10** | **50** | **55** |

**Table S3 Antibiotic resistance genes, IS*Aba1,* IS*Aba125*, and characterization of *bla*_NDM_ genes**

|  | **Code** | **blaNDM** | **blaNDM-1** | **blaNDM-5** | **blaoxa-23** | **blaoxa-24** | **blaoxa-51** | **blaoxa-58** | **ISAba1** | **ISAba125** |
| --- | --- | --- | --- | --- | --- | --- | --- | --- | --- | --- |
|  |  |  |  |  |  |  |  |  |  |  |
| 1 | AB-120 | Present | Present | Absent | Present | Absent | Present | Present | Present | Present |
| 2 | AB-148 | Present | Present | Absent | Absent | Absent | Present | Present | Present | Present |
| 3 | AB-166 | Present | Present | Absent | Absent | Absent | Present | Present | Present | Present |
| 4 | AB-231 | Present | Present | Absent | Present | Absent | Present | Absent | Absent | Present |
| 5 | AB-245 | Present | Present | Absent | Absent | Absent | Present | Absent | Absent | Present |
| 6 | AB-256 | Present | Present | Absent | Absent | Absent | Present | Absent | Absent | Present |
| 7 | AB-282 | Present | Present | Absent | Absent | Absent | Present | Absent | Absent | Present |
| 8 | CR01 | Present | Present | Absent | Present | Absent | Present | Absent | Present | Present |
| 9 | CR04 | Present | Present | Absent | Present | Absent | Present | Absent | Present | Present |
| 10 | CR06 | Present | Present | Absent | Present | Absent | Present | Absent | Present | Present |
| 11 | CR16 | Present | Present | Absent | Present | Absent | Present | Absent | Present | Present |
| 12 | CR18 | Present | Present | Absent | Present | Absent | Present | Absent | Present | Present |
| 13 | CR23 | Present | Present | Absent | Present | Absent | Present | Absent | Absent | Present |
| 14 | CR24 | Present | Present | Absent | Present | Absent | Present | Absent | Present | Present |
| 15 | CR25 | Present | Present | Absent | Present | Absent | Present | Absent | Present | Present |
| 16 | CR28 | Present | Present | Absent | Present | Absent | Present | Absent | Present | Absent |
| 17 | CR29 | Present | Present | Absent | Present | Absent | Present | Absent | Present | Present |
| 18 | CR32 | Present | Present | Absent | Present | Absent | Present | Absent | Present | Present |
| 19 | CR35 | Present | Present | Absent | Present | Absent | Present | Absent | Present | Present |
| 20 | CR40 | Present | Present | Absent | Present | Absent | Present | Absent | Present | Present |
| 21 | CR42 | Present | Present | Absent | Present | Absent | Present | Present | Present | Present |
| 22 | CR43 | Present | Present | Absent | Present | Absent | Present | Absent | Present | Present |
| 23 | CR45 | Present | Present | Absent | Present | Absent | Present | Absent | Present | Present |
| 24 | CR46 | Present | Present | Absent | Present | Absent | Present | Absent | Present | Present |
| 25 | CR47 | Present | Present | Absent | Present | Absent | Present | Absent | Present | Absent |
| 26 | CR48 | Present | Present | Absent | Present | Absent | Present | Absent | Present | Present |
| 27 | CR50 | Present | Present | Absent | Present | Absent | Present | Absent | Present | Present |
| 28 | CR51 | Present | Present | Absent | Absent | Absent | Present | Present | Present | Present |
| 29 | CR52 | Present | Present | Absent | Present | Absent | Present | Absent | Present | Present |
| 30 | CR54 | Present | Present | Absent | Absent | Absent | Present | Present | Present | Present |
| 31 | CR59 | Present | Present | Absent | Present | Absent | Present | Absent | Present | Present |
| 32 | CR60 | Present | Present | Absent | Present | Absent | Present | Absent | Present | Present |
| 33 | CR61 | Present | Present | Absent | Present | Absent | Present | Absent | Present | Present |
| 34 | CR62 | Present | Present | Absent | Present | Absent | Present | Absent | Present | Present |
| 35 | CR64 | Present | Present | Absent | Present | Absent | Present | Absent | Present | Present |
| 36 | CR65 | Present | Present | Absent | Present | Absent | Present | Absent | Present | Present |
| 37 | CR66 | Present | Present | Absent | Present | Absent | Present | Absent | Present | Present |
| 38 | CR67 | Present | Present | Absent | Present | Absent | Present | Absent | Present | Present |
| 39 | CR69 | Present | Present | Absent | Present | Absent | Present | Present | Present | Absent |
| 40 | CR70 | Present | Present | Absent | Absent | Absent | Present | Present | Present | Present |
| 41 | CR75 | Present | Present | Absent | Present | Absent | Present | Absent | Present | Present |
| 42 | CR76 | Present | Present | Absent | Present | Absent | Present | Absent | Present | Present |
| 43 | CR79 | Present | Present | Absent | Present | Absent | Present | Absent | Present | Present |
| 44 | CR80 | Present | Present | Absent | Present | Absent | Present | Absent | Absent | Absent |
| 45 | CR85 | Present | Present | Absent | Present | Absent | Present | Absent | Present | Present |
| 46 | CR87 | Present | Present | Absent | Present | Absent | Present | Absent | Present | Present |
| 47 | CR90 | Present | Present | Absent | Present | Absent | Present | Absent | Present | Present |
| 48 | CR67-07 | Present | Absent | Present | Present | Absent | Present | Absent | Present | Absent |
| 49 | CR67-17 | Present | Present | Absent | Present | Absent | Present | Absent | Present | Absent |
| 50 | CR67-18 | Present | Absent | Present | Present | Absent | Present | Absent | Present | Present |
| 51 | CR67-41 | Present | Absent | Present | Present | Absent | Present | Absent | Present | Absent |
| 52 | CR67-58 | Present | Absent | Present | Present | Absent | Present | Absent | Present | Absent |
| 53 | CR67-72 | Present | Present | Absent | Present | Absent | Present | Present | Present | Present |
| 54 | CR67-91 | Present | Absent | Present | Present | Absent | Present | Absent | Present | Absent |
| 55 | CR67-92 | Present | Absent | Present | Present | Absent | Present | Absent | Present | Absent |
|  |  | **55** | **49** | **6** | **47** | **0** | **55** | **9** | **49** | **45** |

**Table S4 Sequencing identity of blaNDM variant genes from blaNDM positive *A. baumannii* from Chiangrai Prachanukroh Hospital**

| Code | Gene type | Query cover | %identity | sequenced length | reference accession numbers |  |
| --- | --- | --- | --- | --- | --- | --- |
|  |  |  |  |  |  |  |
| AB-120 | NDM-1 | 99 | 100 | 579 | [MH211127.1](https://www.ncbi.nlm.nih.gov/nucleotide/MH211127.1?report=genbank&log$=nucltop&blast_rank=6&RID=50Y3UGV0014) |  |
| AB-148 | NDM-1 | 100 | 100 | 519 | [CP104447.1](https://www.ncbi.nlm.nih.gov/nucleotide/CP104447.1?report=genbank&log$=nucltop&blast_rank=1&RID=519WS16M016) |  |
| AB-166 | NDM-1 | 100 | 100 | 537 | [CP104447.1](https://www.ncbi.nlm.nih.gov/nucleotide/CP104447.1?report=genbank&log$=nucltop&blast_rank=1&RID=519YXT6Y016) |  |
| AB-231 | NDM-1 | 100 | 100 | 562 | [CP029610.1](https://www.ncbi.nlm.nih.gov/nucleotide/CP029610.1?report=genbank&log$=nucltop&blast_rank=5&RID=51A15ARA014) |  |
| AB-245 | NDM-1 | 97 | 100 | 599 | [CP184053.1](https://www.ncbi.nlm.nih.gov/nucleotide/CP184053.1?report=genbank&log$=nucltop&blast_rank=1&RID=51A33TWN014) |  |
| AB-256 | NDM-1 | 100 | 100 | 549 | [CP104447.1](https://www.ncbi.nlm.nih.gov/nucleotide/CP104447.1?report=genbank&log$=nucltop&blast_rank=1&RID=51A4VXW4016) |  |
| AB-282 | NDM-1 | 100 | 100 | 519 | [CP086673.1](https://www.ncbi.nlm.nih.gov/nucleotide/CP086673.1?report=genbank&log$=nucltop&blast_rank=8&RID=51A8JUJM014) |  |
| CR01 | NDM-1 | 100 | 100 | 500 | [CP104447.1](https://www.ncbi.nlm.nih.gov/nucleotide/CP104447.1?report=genbank&log$=nucltop&blast_rank=2&RID=58NS99N8014) |  |
| CR04 | NDM-1 | 100 | 100 | 500 | [MH211127.1](https://www.ncbi.nlm.nih.gov/nucleotide/MH211127.1?report=genbank&log$=nucltop&blast_rank=3&RID=58RHNYK6014) |  |
| CR06 | NDM-1 | 100 | 100 | 509 | [CP086673.1](https://www.ncbi.nlm.nih.gov/nucleotide/CP086673.1?report=genbank&log$=nucltop&blast_rank=8&RID=58S62PS9014) |  |
| CR16 | NDM-1 | 100 | 100 | 500 | [OL544134.1](https://www.ncbi.nlm.nih.gov/nucleotide/OL544134.1?report=genbank&log$=nucltop&blast_rank=35&RID=58SWD7VN014) |  |
| CR18 | NDM-1 | 100 | 100 | 510 | [MH257689.1](https://www.ncbi.nlm.nih.gov/nucleotide/MH257689.1?report=genbank&log$=nucltop&blast_rank=32&RID=58TRPHH4014) |  |
| CR23 | NDM-1 | 100 | 100 | 468 | [MH257689.1](https://www.ncbi.nlm.nih.gov/nucleotide/MH257689.1?report=genbank&log$=nucltop&blast_rank=35&RID=58TXSHDW014) |  |
| CR24 | NDM-1 | 100 | 100 | 319 | [LC723920.1](https://www.ncbi.nlm.nih.gov/nucleotide/LC723920.1?report=genbank&log$=nucltop&blast_rank=3&RID=58U1NTRH014) |  |
| CR25 | NDM-1 | 100 | 100 | 490 | [MG436908.1](https://www.ncbi.nlm.nih.gov/nucleotide/MG436908.1?report=genbank&log$=nucltop&blast_rank=18&RID=58UW30P1016) |  |
| CR28 | NDM-1 | 100 | 100 | 380 | [MH257689.1](https://www.ncbi.nlm.nih.gov/nucleotide/MH257689.1?report=genbank&log$=nucltop&blast_rank=4&RID=58UZ4WB2014) |  |
| CR29 | NDM-1 | 100 | 100 | 559 | [MK834311.1](https://www.ncbi.nlm.nih.gov/nucleotide/MK834311.1?report=genbank&log$=nucltop&blast_rank=13&RID=58V16SMS014) |  |
| CR32 | NDM-1 | 100 | 100 | 509 | [LC723920.1](https://www.ncbi.nlm.nih.gov/nucleotide/LC723920.1?report=genbank&log$=nucltop&blast_rank=13&RID=58VKYSZ0016) |  |
| CR35 | NDM-1 | 100 | 100 | 579 | [MH211130.1](https://www.ncbi.nlm.nih.gov/nucleotide/MH211130.1?report=genbank&log$=nucltop&blast_rank=3&RID=58VPU4ZC014) |  |
| CR40 | NDM-1 | 100 | 100 | 569 | [MH211130.1](https://www.ncbi.nlm.nih.gov/nucleotide/MH211130.1?report=genbank&log$=nucltop&blast_rank=3&RID=58VUK96S014) |  |
| CR42 | NDM-1 | 100 | 100 | 569 | [MN422296.1](https://www.ncbi.nlm.nih.gov/nucleotide/MN422296.1?report=genbank&log$=nucltop&blast_rank=10&RID=58VX0KP6014) |  |
| CR43 | NDM-1 | 100 | 100 | 479 | [CP128795.1](https://www.ncbi.nlm.nih.gov/nucleotide/CP128795.1?report=genbank&log$=nucltop&blast_rank=14&RID=58VYM04M014) |  |
| CR45 | NDM-1 | 100 | 99.77 | 439 | [MH257689.1](https://www.ncbi.nlm.nih.gov/nucleotide/MH257689.1?report=genbank&log$=nucltop&blast_rank=33&RID=58W374JH016) |  |
| CR46 | NDM-1 | 100 | 100 | 569 | [MH211127.1](https://www.ncbi.nlm.nih.gov/nucleotide/MH211127.1?report=genbank&log$=nucltop&blast_rank=8&RID=58W5A55H014) |  |
| CR47 | NDM-1 | 100 | 100 | 539 | [MH211127.1](https://www.ncbi.nlm.nih.gov/nucleotide/MH211127.1?report=genbank&log$=nucltop&blast_rank=3&RID=58W9ASB1016) |  |
| CR48 | NDM-1 | 98 | 99.82 | 559 | [CP128795.1](https://www.ncbi.nlm.nih.gov/nucleotide/CP128795.1?report=genbank&log$=nucltop&blast_rank=12&RID=58WB9P9A014) |  |
| CR50 | NDM-1 | 100 | 100 | 490 | [MH211127.1](https://www.ncbi.nlm.nih.gov/nucleotide/MH211127.1?report=genbank&log$=nucltop&blast_rank=3&RID=58WF3STY016) |  |
| CR51 | NDM-1 | 100 | 100 | 579 | [CP184053.1](https://www.ncbi.nlm.nih.gov/nucleotide/CP184053.1?report=genbank&log$=nucltop&blast_rank=1&RID=58WHCE3H014) |  |
| CR52 | NDM-1 | 100 | 100 | 410 | [MH257689.1](https://www.ncbi.nlm.nih.gov/nucleotide/MH257689.1?report=genbank&log$=nucltop&blast_rank=3&RID=58WJZEU7016) |  |
| CR54 | NDM-1 | 98 | 100 | 579 | [MK372386.1](https://www.ncbi.nlm.nih.gov/nucleotide/MK372386.1?report=genbank&log$=nucltop&blast_rank=19&RID=58WK72AJ014) |  |
| CR59 | NDM-1 | 100 | 100 | 559 | [MK834311.1](https://www.ncbi.nlm.nih.gov/nucleotide/MK834311.1?report=genbank&log$=nucltop&blast_rank=8&RID=58WP9C57014) |  |
| CR60 | NDM-1 | 100 | 100 | 539 | [OL544134.1](https://www.ncbi.nlm.nih.gov/nucleotide/OL544134.1?report=genbank&log$=nucltop&blast_rank=16&RID=58WPABJ8014) |  |
| CR61 | NDM-1 | 100 | 100 | 509 | [MH211127.1](https://www.ncbi.nlm.nih.gov/nucleotide/MH211127.1?report=genbank&log$=nucltop&blast_rank=3&RID=58WSJ95U014) |  |
| CR62 | NDM-1 | 100 | 100 | 579 | [MH211127.1](https://www.ncbi.nlm.nih.gov/nucleotide/MH211127.1?report=genbank&log$=nucltop&blast_rank=3&RID=58WSJ95U014) |  |
| CR64 | NDM-1 | 100 | 100 | 579 | [CP101277.1](https://www.ncbi.nlm.nih.gov/nucleotide/CP101277.1?report=genbank&log$=nucltop&blast_rank=4&RID=58WVE36U014) |  |
| CR65 | NDM-1 | 100 | 100 | 559 | [MN422296.1](https://www.ncbi.nlm.nih.gov/nucleotide/MN422296.1?report=genbank&log$=nucltop&blast_rank=13&RID=58WVMCTB016) |  |
| CR66 | NDM-1 | 96 | 100 | 649 | [LC521853.1](https://www.ncbi.nlm.nih.gov/nucleotide/LC521853.1?report=genbank&log$=nucltop&blast_rank=12&RID=58WXREK7014) |  |
| CR67 | NDM-1 | 100 | 100 | 559 | [MH211127.1](https://www.ncbi.nlm.nih.gov/nucleotide/MH211127.1?report=genbank&log$=nucltop&blast_rank=3&RID=58WXZC1X014) |  |
| CR69 | NDM-1 | 100 | 100 | 569 | [CP086673.1](https://www.ncbi.nlm.nih.gov/nucleotide/CP086673.1?report=genbank&log$=nucltop&blast_rank=13&RID=58X2H8KJ016) |  |
| CR70 | NDM-1 | 100 | 100 | 569 | [MH211127.1](https://www.ncbi.nlm.nih.gov/nucleotide/MH211127.1?report=genbank&log$=nucltop&blast_rank=8&RID=58X2P8HZ014) |  |
| CR75 | NDM-1 | 100 | 100 | 569 | [MK834311.1](https://www.ncbi.nlm.nih.gov/nucleotide/MK834311.1?report=genbank&log$=nucltop&blast_rank=9&RID=58X4JTKC014) |  |
| CR76 | NDM-1 | 99 | 100 | 599 | [MH211130.1](https://www.ncbi.nlm.nih.gov/nucleotide/MH211130.1?report=genbank&log$=nucltop&blast_rank=3&RID=58X4PF10014) |  |
| CR79 | NDM-1 | 100 | 100 | 569 | [MH211127.1](https://www.ncbi.nlm.nih.gov/nucleotide/MH211127.1?report=genbank&log$=nucltop&blast_rank=3&RID=58X7E45A014) |  |
| CR80 | NDM-1 | 100 | 100 | 510 | [LC521853.1](https://www.ncbi.nlm.nih.gov/nucleotide/LC521853.1?report=genbank&log$=nucltop&blast_rank=7&RID=58XK9K35014) |  |
| CR85 | NDM-1 | 100 | 100 | 569 | [CP101277.1](https://www.ncbi.nlm.nih.gov/nucleotide/CP101277.1?report=genbank&log$=nucltop&blast_rank=4&RID=58XKGUZ1014) |  |
| CR87 | NDM-1 | 100 | 100 | 549 | [MH211127.1](https://www.ncbi.nlm.nih.gov/nucleotide/MH211127.1?report=genbank&log$=nucltop&blast_rank=3&RID=58XNSBPR014) |  |
| CR90 | NDM-1 | 100 | 100 | 579 | [CP104447.1](https://www.ncbi.nlm.nih.gov/nucleotide/CP104447.1?report=genbank&log$=nucltop&blast_rank=2&RID=58XNWVMK014) |  |
| CR67-07 | NDM-5 | 100 | 99.82 | 561 | [OP806925.1](https://www.ncbi.nlm.nih.gov/nucleotide/OP806925.1?report=genbank&log$=nucltop&blast_rank=1&RID=58XS7J5Z014) |  |
| CR67-17 | NDM-1 | 97 | 100 | 467 | [MH211127.1](https://www.ncbi.nlm.nih.gov/nucleotide/MH211127.1?report=genbank&log$=nucltop&blast_rank=3&RID=58XTUVPF014) |  |
| CR67-18 | NDM-5 | 100 | 100 | 449 | [PX308688.1](https://www.ncbi.nlm.nih.gov/nucleotide/PX308688.1?report=genbank&log$=nucltop&blast_rank=9&RID=58XU4BGZ016) |  |
| CR67-41 | NDM-5 | 100 | 100 | 569 | [OP806925.1](https://www.ncbi.nlm.nih.gov/nucleotide/OP806925.1?report=genbank&log$=nucltop&blast_rank=4&RID=58XWKKJ5014) |  |
| CR67-58 | NDM-5 | 100 | 100 | 569 | [OP806925.1](https://www.ncbi.nlm.nih.gov/nucleotide/OP806925.1?report=genbank&log$=nucltop&blast_rank=1&RID=58XWUJUV014) |  |
| CR67-72 | NDM-1 | 100 | 100 | 559 | [CP184053.1](https://www.ncbi.nlm.nih.gov/nucleotide/CP184053.1?report=genbank&log$=nucltop&blast_rank=1&RID=58XYRBJD014) |  |
| CR67-91 | NDM-5 | 100 | 100 | 559 | [PX308688.1](https://www.ncbi.nlm.nih.gov/nucleotide/PX308688.1?report=genbank&log$=nucltop&blast_rank=8&RID=58XYX88S014) |  |
| CR67-92 | NDM-5 | 100 | 100 | 569 | [OP806925.1](https://www.ncbi.nlm.nih.gov/nucleotide/OP806925.1?report=genbank&log$=nucltop&blast_rank=1&RID=58Y0Z4KB014) |  |
|  |  |  |  |  |  |  |

**Table S5 Percent resistance phenotype of *Acinetobacter baumannnii* carried other Oxacillinase genes co-occerence with blaNDM gene.**

| **Genotype** | **N** | **% Resistance** | | | | | |
| --- | --- | --- | --- | --- | --- | --- | --- |
|  |  | **CARBAPENEMS** | | **CEPHEMS** | | | |
|  |  | Meropemens(MEM) | Imipinem (IPM) | Cefotaxime (CTX) | Ceftazidime (CAZ) | Ceftriaxone (CRO) | Cefepime (FEP) |
| *bla_NDM-1_* *- bla*_OXA-23_ | 41 | 100 | 100 | 100 | 100 | 100 | 100 |
| *bla_NDM-1_* *- bla*_OXA-58_ | 9 | 100 | 100 | 100 | 100 | 100 | 100 |
| *bla_NDM-5_* *- bla*_OXA-23_ | 6 | 100 | 100 | 100 | 100 | 100 | 100 |
|  |  |  |  |  |  |  |  |
|  |  |  |  |  |  |  |  |
|  |  |  |  |  |  |  |  |
|  |  |  |  |  |  |  |  |
|  |  |  |  |  |  |  |  |
